# Utility of phrenic nerve ultrasound in amyotrophic lateral sclerosis

**DOI:** 10.1007/s13760-020-01531-y

**Published:** 2020-11-02

**Authors:** Cezar Thomas Suratos, Naoko Takamatsu, Hiroki Yamazaki, Yusuke Osaki, Tatsuya Fukumoto, Hiroyuki Nodera, Yuishin Izumi

**Affiliations:** 1 Department of Neurology, Tokushima University Hospital, Tokushima, Japan; 2 Department of Neurology, Kanazawa Medical University, Kanazawa, Japan; 3 Department of Neurology, Vihara Hananosato Hospital, Miyoshi, Japan

**Keywords:** Amyotrophic lateral sclerosis, Phrenic nerve, Peripheral nerve ultrasound, Peripheral nerve size

## Abstract

Amyotrophic lateral sclerosis (ALS) is a neurodegenerative disorder affecting the upper and lower motor neurons causing progressive weakness. It eventually involves the diaphragm which leads to respiratory paralysis and subsequently death. Phrenic nerve (PN) conduction studies and diaphragm ultrasound has been studied and correlated with pulmonary function tests in ALS patients. However, PN ultrasonography has not been employed in ALS. This study aims to sonographically evaluate the morphologic appearance of the PN of ALS patients. Thirty-eight ALS patients and 28 normal controls referred to the neurophysiology laboratory of two institutions were retrospectively included in the study. Baseline demographic and clinical variables such as disease duration, ALS Functional Rating Scale-Revised score, and ALS region of onset were collected. Ultrasound was used to evaluate the PN cross-sectional area (CSA) of ALS and control subjects. The mean PN CSA of ALS patients were 1.08 ± 0.39 mm on the right and 1.02 ± 0.34 mm on the left. The PN CSA of ALS patients were significantly decreased compared to controls (*p* value < 0.00001). The PN CSA of ALS patients was not correlated to any of the demographic and clinical parameters tested. This study demonstrates that ALS patients have a smaller PN size compared to controls using ultrasonography.

## Introduction

Amyotrophic lateral sclerosis (ALS) is a neurodegenerative disorder predominantly affecting the upper and lower motor neurons causing progressive weakness and muscle wasting [1]. Weakness eventually involving the diaphragm leads to respiratory paralysis and subsequently death in ALS [1, 2]. As a surrogate marker for respiratory function, diaphragm ultrasound has been employed in ALS in several studies [3–6]. Diaphragm thickness was observed to be reduced in ALS and it is correlated with measures of respiratory function [2–5]. Phrenic nerve (PN) motor conduction studies have also been used to evaluate the respiratory function and have been observed to detect latent respiratory dysfunction as well as predict survival in ALS [7, 8].

Recently, peripheral nerve ultrasound has been investigated in ALS for diagnostic and monitoring purposes [2]. Measurements of nerve cross-sectional area (CSA) and diameter have been employed and decreased nerve caliber was consistently reported in different nerves [2, 9–12]. However, no consensus has yet been achieved as to which nerves are specific and sensitive for ALS diagnosis and disease progression monitoring [2].

In a postmortem study of 11 ALS patients, the majority of large myelinated fibers were lost with some acute axonal degeneration seen in the PN [13]. To demonstrate in vivo changes, this study aims to determine if a pure motor nerve such as the phrenic nerve would sonographically show evidence of decreased nerve caliber similar to other peripheral nerves in ALS patients.

## Methods

Subjects with a final diagnosis of ALS based on the updated Awaji criteria were identified from the list of patients referred to the neurophysiology laboratories of Tokushima University Hospital and Vihara Hananosato Hospital from 2018 to 2019 [14]. Patients with complete data on ultrasonographic PN CSA measurements were retrospectively included. Patients were seen on an outpatient basis and without overt respiratory distress. Baseline demographic data including age, sex, ALS disease duration, ALS region of onset, and most recent ALS Functional Rating Scale-Revised (ALSFRS-R) score were obtained. Normal control CSA values of the PN were obtained from the hospital staff and residents of an elderly facility which did not exhibit any neurological symptoms.

Ultrasonography was done by two experienced authors, NT and HY. All normal controls were done by NT and ALS patients were done by NT and HY in a non-randomized non-blinded manner. To account for other sources of measurement error, the patients were divided into two cohorts based on the ultrasonographer and the demographic and clinical characteristics were compared. There was a significant difference in terms of age, disease duration and ALSFRS-R score between the two groups. However, the PN CSA measurements done by the two ultrasonographers were similar between the two groups. With multivariate analysis, it can be noted that age, disease duration and ALSFRS-R scores have F-statistic values less than 5, which is considered very low, hence, the authors believe that these have little relationship with PN CSA measurements. Nevertheless, these are considered confounding variables in this study.

LOGIQ e Premium device with a 12-MHz linear array transducer was used for sonographic analysis. All ultrasonographic analyses were done in the supine position. Axial images of the PN were obtained by locating the scalene muscles at the interscalene triangle. The PN is measured as it crosses over the anterior scalene muscle, coursing towards the internal jugular vein and common carotid artery on the medial side of the neck [15]. The CSA of the PN was measured by manually tracing the area within the nerve’s hyperechoic rim using the trace function on the machine. The right hemidiaphragm (HD) was visualized by placing the probe on the right intercostal space between the anterior axillary line and the mid-axillary line. The HD was visibly outlined by two parallel lines, the pleural line on top and the peritoneal line below. The HD width was measured by taking the distance from the pleural line to the peritoneal line. Measurements were made during relaxed position, labeled as diaphragm width at rest (DRest), during maximal inspiration, labeled as inspiratory width (DIns), and during maximal expiration, labeled as expiratory width (DExp). The thickening ratio was calculated as the ratio of DIns to DExp [3, 16].

This study was approved by the institutional review board of two institutions and the subjects gave written informed consent at the time of testing.

## Statistical analysis

Statistical analysis was conducted by one of the authors. R statistical software (R Foundation for Statistical Computing, Vienna, Austria) was used for all statistical analyses and statistical significance was set at *p* < 0.05. Descriptive statistics were used for the characterization of the sample. Multivariate analysis was done to determine the correlation of the demographic variables to the PN CSA. Linear regression analysis using the Kendall correlation coefficient (tau) was used to correlate PN CSAs with clinical parameters. Computation for correlation was likewise done on the right PN CSA and right HD measurements. Wilcoxon signed-rank test or Kruskal–Wallis rank-sum test were used where appropriate to check for the difference in paired and unpaired samples.

## Results

There were 38 ALS patients and 28 control subjects included in the study. According to the updated Awaji criteria 9 patients were classified as definite ALS, 14 were probable ALS, 14 were probable-laboratory supported ALS, and 1 was possible ALS. Based on the initial region of onset, 24 were upper limb-onset, 5 were lower limb-onset, and 9 were bulbar-onset. The mean disease duration from onset to evaluation was 26.16 months (Interquartile range 7 months, 37 months) and the mean ALSFRS-R score was 38.41 ± 6.87.

Table 1 shows the demographic characteristics of the subjects. Both groups were similar in terms of age, sex, height, weight, and body mass index (BMI). Although no significant difference was seen between the demographic variables of the two groups, it can be observed that ALS patients have a higher mean age and height and have more male subjects. A multivariate analysis of the demographic variables showed that in normal controls, age (*p* < 0.001) and height (*p* < 0.001) were directly correlated to the right PN. However, the adjusted R^2^ for the regression model is only 0.37, suggesting that age and height contribute to only 37% of the variability in the measurements of the right PN CSA. Additionally, the F-statistic is only 9.06, which is quite small to explain a relationship between age and height and the right PN CSA. The left PN of normal subjects and both the right and left PN of ALS patients were not correlated to the demographic characteristics.

**Table 1:** Demographic characteristics of the control subjects and ALS patients

Variable	Control	ALS	*p* value^a^
Number	28	38	
Age years, mean ± SD (range, years)	59.61 ± 24.45 (19–93)	64.71 ± 12.76 (31–87)	0.53
Sex (M:F)	10:18	23:15	0.08^b^
Height cm, mean ± SD (range, cm)	157.75 ± 7.36 (140–171)	161.50 ± 8.76 (138.6–178)	0.08
Weight kg, mean ± SD (range, kg)	56.89 ± 13.26 (35–96)	56.41 ± 11.15 (34.4–81.5)	0.98
BMI kg/m^2^, mean ± SD (range, kg/m^2^)	22.72 ± 4.40 (16.23–36.58)	21.52 ± 3.19 (14.17–26.89)	0.54

*BMI* Body Mass Index

^a^Wilcoxon signed-rank test

^b^*Χ*^2^ test for equality of proportions

**Fig. 1:**
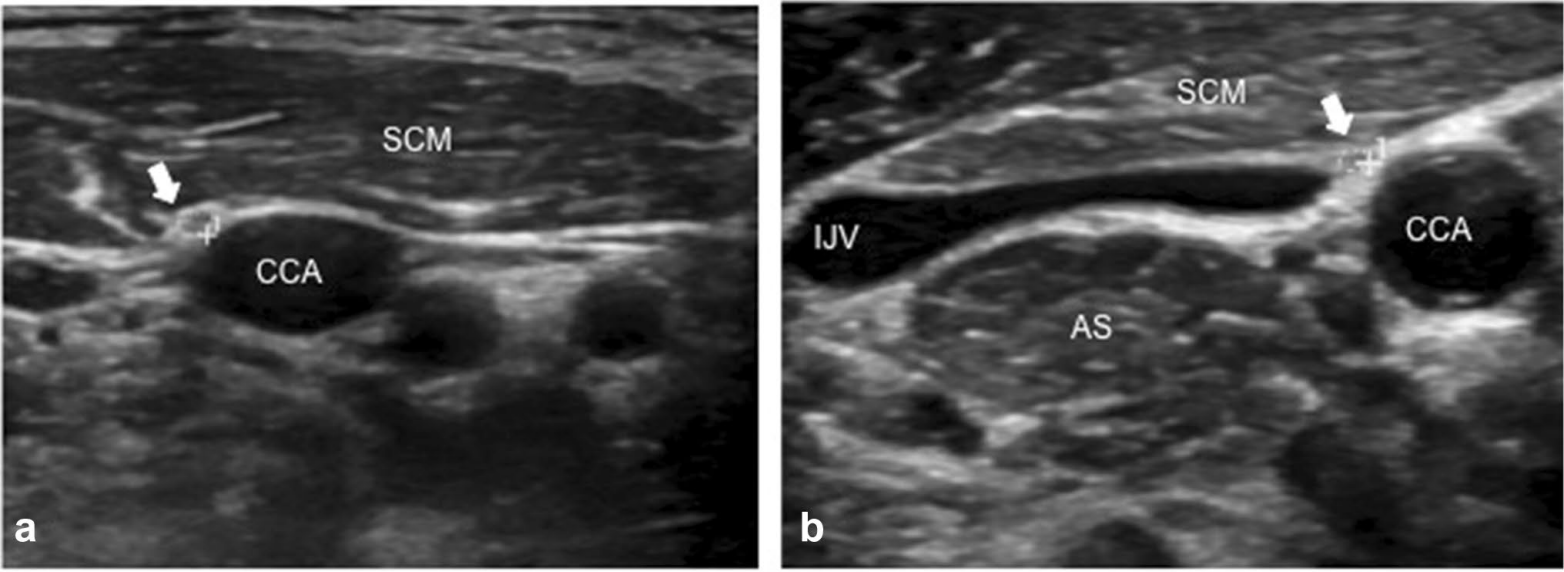
The PN, marked by the solid white arrows, are seen here as it courses above the anterior scalene muscle coursing towards the common carotid artery. **a** The PN CSA of the normal subject was 1.4 mm^2^. **b** The PN CSA was 0.6 mm^2^ for the ALS patient. *AS* anterior scalene muscle, *CCA* common carotid artery, *IJV* internal jugular vein, *SCM* sternocleidomastoid

Figure 1 shows the representative sonographic images of the PN in a control and an ALS subject. The mean and standard deviation values for the PN CSA and different HD width measurements are seen in Table 2. The mean right and left PN CSA values of the ALS patients were significantly different from the normal controls. For the HD measurements, it must be noted that some data were missing. Only a total of 18 patients had DExp and DIns measurements while 20 patients had DRest measurements.

**Table 2:** Clinical characteristics of the control subjects and ALS patients

Variable	Control	ALS	*p* value^a^
Number	28	38	
Right PN CSA mm^2^, mean ± SD (range, mm^2^)	1.62 ± 0.45 (1.010–2.840)	1.08 ± 0.39 (0.52–2.05)	< 0.00001
Left PN CSA mm^2^, mean ± SD (range, mm^2^)	1.39 ± 0.31 (1.010–2.150)	1.02 ± 0.34 (0.45–1.69)	< 0.00001
DRest mm, mean ± SD (range, mm)	N/A	1.34 ± 0.38 (*n* = 20) (0.5–2.0)	
DExp mm, mean ± SD (range, mm)	N/A	1.21 ± 0.37 (*n* = 18) (0.5–2.1)	
DIns mm, mean ± SD (range, mm)	N/A	2.03 ± 0.77 (*n* = 18) (0.6–4.0)	
Thickening ratio	N/A	1.66 ± 0.27 (*n* = 18) (1.2–2.2)	

*DRest* diaphragm width at rest, *DExp* expiratory diaphragm width, *DIns* inspiratory diaphragm width, *thickening ratio* DIns/Dexp

^a^Wilcoxon signed-rank test

**Fig. 2:**
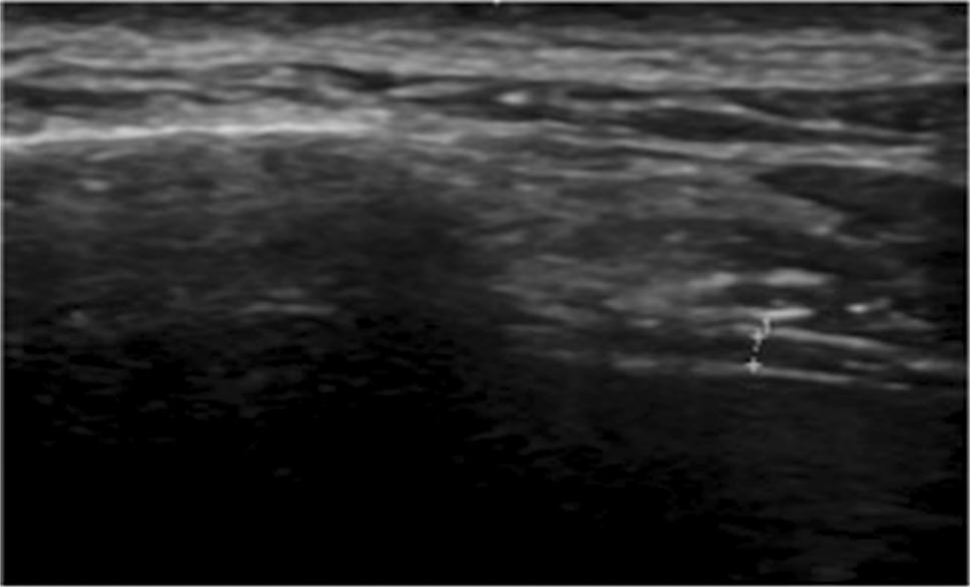
Representative image of the right HD of an ALS patient taken at rest. The crosshairs mark the pleural line (top) and the peritoneal line (bottom) with the DRest measuring 0.9 mm

The right HD at the rest of an ALS patient is illustrated in Fig. 2. No correlation was seen between the clinical variables and PN CSAs. Both the right and left PN CSAs were not correlated to the disease parameters tested – disease duration, ALSFRS-R score, and ALS region of onset. The right PN CSA was not correlated to DExp, DIns, DRest or thickening ration of the right HD.

**Fig. 3:**
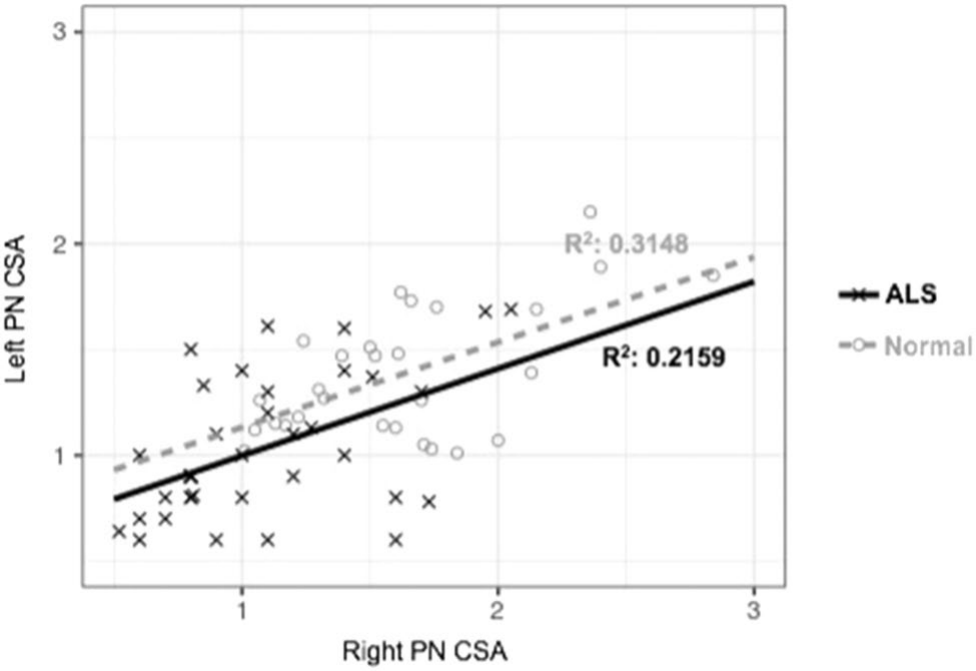
The correlation plots between the right and left PN CSA for both the normal controls and ALS subjects. *R*^2^ adjusted *R* value

The right PN CSA was significantly larger than the left PN CSA among normal controls only (*p* < 0.01). The right and left PN CSA were not significantly different from each other in ALS patients. Additionally, both the right and left PN CSA were directly correlated to each other in both normal and ALS subjects (*p* < 0.05). The correlation plot is shown in Fig. 3.

## Discussion

This study demonstrated that the PN of ALS patients were significantly smaller than normal controls. As reported in ultrasound studies of other peripheral nerves, progressive motor neuron loss resulting in axonal degeneration is implicated as the cause of smaller nerve caliber observed in ALS [2, 9, 10]. Congruent to our findings, a postmortem study of the PN of 11 ALS patients showed uniform loss of two-thirds of large myelinated axons from the proximal to the distal region [13]. Apart from axonal loss, significant distal axonal atrophy of the PN was seen when compared with controls [13].

The multivariate analysis of the demographic variables showed that age and height were correlated to the right PN CSA of normal controls. In a sonographic analysis of healthy subjects, age was seen to be indirectly correlated to PN CSA but not correlated to height [17]. The correlation with age was attributed by the authors to age-related degeneration of myelinated nerve fibers [17]. However, the PN CSA of ALS subjects were not correlated to age despite having an older population cohort. The age and height of ALS patients were larger than those of normal controls, but this was not statistically significant. The authors attribute the finding of a correlation between age and right PN CSA of normal controls to have a minimal effect due to the low *R*^2^ and *F*-statistic values.

The right PN CSA of normal controls were significantly larger than the left PN CSA. A study among 20 healthy volunteers showed a larger right PN CSA compared to the left but the difference was likewise not significant [18]. The same trend was not seen among ALS subjects where the right was not significantly different from the left PN CSA. Oxidative damage has been suggested as the cause of axonal dysfunction and degradation in ALS [19]. Oxidative stess affecting diffuse cellular processes may explain the diffuse degeneration of both the right and left PN.

Bilateral PN CSA was not correlated to any of the clinical variables. There was no correlation between PN CSA and disease duration. This may suggest that the PN morphology may be affected even at an early time in the disease process. Studies on animal models of motor neuron disease have shown that distal axonal degeneration may precede symptom onset and motor neuron death [19]. However, this pathologic sequence has not been proven in human ALS cases. If the PN indeed shows changes even at an early disease course, a longitudinal study sonographically evaluating the PN may be helpful in this situation. The PN CSA were also not correlated to ALSFRS-R score and initial region of onset. Nerve conduction studies of the PN were not correlated to ALS region of onset, signifying that the loss of motor units was comparable whether the region of onset was bulbar or spinal [20]. The ALSFRS-R score was likewise not correlated to PN conduction studies [7]. These studies along with our study may suggest that the PN CSA and the PN conduction studies are not reliable markers to show changes in relation to the ALSFRS-R score or ALS region of onset.

The mean DExp was 1.21 ± 0.37 mm while the thickening ratio was 1.66 ± 0.27. Unfortunately, HD measurements were not taken from the control group hence statistical comparisons could not be made in this study. A published study has set the reference value of right HD thickness at normal end-expiration at 0.33 ± 0.1 cm or 3.3 mm, and thickening ratio at 1.8 ± 0.5 cm (18 mm) [16]. The mean diaphragm measurements seen in our set of ALS patients is lower by more than half compared to the normal subjects in the abovementioned study, however, a statistical comparison cannot be made. Previous studies have demonstrated that ALS patients have decreased diaphragm thickness or diaphragm thickening ratios compared to controls [3, 4]. More importantly, the diaphragm ultrasound thickening ratio was correlated with respiratory function tests such as forced vital capacity, nasal inspiratory pressure during sniff and maximal voluntary ventilation [5]. These previous reports suggest the use of diaphragm ultrasound in assessing pulmonary function in ALS patients [3, 4].

The limitation of this study is its retrospective design. Measurement bias may have been present due to the observational nature of the study and the lack of evaluation of interrater reliability. The difference observed between the PN CSA of the controls and the ALS patients may have been affected by various confounders during the measurement process. Future research employing a blinded measurement of the PN CSA with inter-rater reliability measurements may help provide stronger proof of PN CSA changes in ALS patients. Additionally, the phrenic nerve ultrasound was not correlated with pulmonary function tests (PFT). Diaphragm ultrasound and PN conduction studies have been demonstrated to be well correlated to PFT in prior studies [3–8]. Hence, future studies may evaluate the value of PN ultrasound to PFT and assess respiratory dysfunction more directly in ALS.

## Conclusion

In conclusion, this study demonstrates that ALS patients have a smaller PN size compared to controls using ultrasonography.

## Abbreviations

ALS: Amyotrophic lateral sclerosis
ALSFRS-R: ALS Functional Rating Scale-Revised
CSA: Cross-sectional area
DExp: Right hemidiaphragm width at expiration;
Dins: Right hemidiaphragm width during maximal inspiration
DRest: Right hemidiaphragm width at rest
HD: Hemidiaphragm
PFT: Pulmonary function test
PN: Phrenic nerve
